# Identification of three prominin homologs and characterization of their messenger RNA expression in *Xenopus laevis* tissues

**Published:** 2011-05-25

**Authors:** Zhou Han, David S. Papermaster

**Affiliations:** Department of Neuroscience, University of Connecticut Health Center, Farmington, CT

## Abstract

**Purpose:**

Prominin is a family of pentaspan transmembrane glycoproteins. They are expressed in various types of cells, including many stem/progenitor cells. Prominin-1 plays an important role in generating and maintaining the structure of the photoreceptors. In this study, we identified three prominin homologs in *Xenopus laevis*, a model animal widely used in vision research, and characterized their messenger RNA (mRNA) expression in selected tissues of this frog.

**Methods:**

Reverse-transcription PCR (RT–PCR) and rapid amplification of cDNA ends (RACE) were used to isolate cDNAs of prominin homologs. Semiquantitative RT–PCR was used to measure the relative expression levels of mRNAs of the three prominin homologs in four *X. laevis* tissues, specifically those of the retina, brain, testis, and kidney. Sequences of prominin homologs were analyzed with bioinformatic software.

**Results:**

We isolated cDNAs of three prominin homologs from *X. laevis* tissues and compared their sequences with previously described prominin-1, 2, and 3 sequences from other species using phylogenetic analysis. Two of these homologs are likely to be the *X. laevis* orthologs of mammalian prominin-1 and 2, respectively, while the third homolog is likely to be the *X. laevis* ortholog of prominin-3, which was only found in nonmammalian vertebrates and the platypus. We identified alternatively spliced exons in mRNAs of all three prominin homologs. Similar to mammalian prominin-1, we found that exons 26b, 27, and 28a of the *X. laevis* prominin-1 gene are alternatively spliced, and that the splice isoforms of mRNA show tissue-specific expression profiles. We found that prominin-1 was the most abundant homolog expressed in the retina, brain, and testis, while prominin-3 was the most abundant homolog in the kidney. The expression level of prominin-2 was the lowest of the three prominin homologs in all four examined tissues of this frog.

**Conclusions:**

Our results suggest that the mRNAs of prominin homologs are expressed in many tissues of *X. laevis,* but differ in their expression levels and mRNA splicing. Prominin-1 is the most abundant of the three prominin homologs expressed in the frog retina.

## Introduction

The prominins are a family of pentaspan transmembrane glycoproteins [1-5]. The first member, identified as prominin-1, was originally cloned from murine kidney epithelial cells [6]. It was later characterized as an immunological determinant of hematopoietic and neuroepithelial stem/progenitor cells [6-8], referred to as CD133 or AC133 [2,7]. Prominin-1 is also expressed in embryonic stem cells [9], cancer cells [10-14], some epithelial cells [15-17], glial cells [18], and rod photoreceptors [19,20]. Prominin-1 is specifically associated with membrane protrusions such as microvilli, filopodia, and lamellipodia [17,21]. Maw et al. [19] reported that a frameshift mutation (PROML1_1878_, now designated as c.1841delG) in prominin-1 that results in the premature termination of translation causes autosomal recessive retinal degeneration in a consanguineous Indian pedigree. Another mutation, R373C, of human prominin-1 was reported to cause autosomal dominant macular degeneration in patients [22,23]. Permanyer et al. identified a third mutation, c.869delG, of human prominin-1 that causes autosomal recessive retinitis pigmentosa [24]. Zacchigna et al. found that knockout of prominin-1 in mice causes slow retinal degeneration [20]. The specific localization of prominin-1 in murine rod photoreceptors and the pathological effect caused by its mutations in humans and mice suggest that it may be involved in the generation or maintenance of photoreceptor outer segment structure.

A second member of the prominin family, prominin-2, shares many features with prominin-1, including a predicted pentatransmembrane topology [3], preferential subcellular localization to membrane protrusions [25], binding to cholesterol, and association with vesicles released into the body fluids [16,25-27]. However, the expression profile of prominin-2 is different from that of prominin-1 in many tissues [3,28,29], and prominin-2 is not detected in the mouse eye [3].

The prominin-1 gene is alternatively spliced in human and mouse tissues, producing multiple splice isoforms of mRNA [2,18,30-32]. Kemper et al. showed that the expression of these splice isoforms is regulated in mouse tissues, based on the fact that these splice isoforms differ in their expression profiles or levels in analyzed tissues [30]. A region that encodes the C-terminus of the human and mouse prominin-1 is extensively spliced [2,31,32]. The significance of the alternative splicing of prominin-1, especially at the C-terminus, is unknown. PDZ (Postsynaptic density protein, PSD-95; Discs large tumor suppressor, DlgA; Zonula occludens-1 protein, ZO-1)-binding motifs have been identified in certain splice isoforms of human and mouse prominin-1 [31]. It was postulated that these splice isoforms interact with different partners and therefore participate in different cellular functions [31-33].

Our goal is to study the function of prominin homologs in the retina of *X. laevis*, in which photoreceptor outer segment membrane synthesis and structure has been investigated by retinal cell biologists for decades [34,35], and for which genetic manipulation methods are available [36,37]. Here, we present the results of the isolation of cDNAs of three prominin homologs in *X. laevis*, comparisons with prominin homologs from other species by phylogenetic analysis, characterization of alternative splicing of prominin-1, and analysis of mRNA expression levels of three prominin homologs by semiquantitative RT–PCR.

## Methods

### Animals

Adult *X. laevis* and *X. tropicalis* were purchased from *Xenopus* Express, Brooksville, FL. The animals were kept in an environment of artificially controlled 12 h:12 h light-dark cycle and constant temperature of 18 °C. All experiments were conducted in accordance with the Animal Care and Use Committee of the University of Connecticut Health Center and the ARVO Statement for the Use of Animals in Ophthalmic and Vision Research.

### Cloning of *Xenopus laevis* prominin-1 cDNA

A protein versus translated nucleotide BLAST search was performed against the Sanger Institute *X. laevis* expressed sequence tag (EST) database, using the protein sequence of human prominin-1 (GenBank accession number: NP_006008) as a query. Nested reverse-transcription (RT)-PCR was performed to isolate a partial cDNA from an *X. laevis* (adult) retinal cDNA library (a gift from Dr. Joseph C. Besharse, Medical College of Wisconsin, WI) [38]. Full-length cDNAs were subsequently isolated by performing 5′ and 3′ rapid amplification of cDNA ends (RACE) using cDNA synthesized from *X. laevis* retina total RNA as a template [39]. Sequences of primers used in nested RT–PCR and RACE are provided in Table 1. The isolated cDNAs were cloned into pCR 2.1 TOPO cloning vector (Invitrogen, Carlsbad, CA) and sequenced using the dye-termination method [40].

**Table 1:** Primers used in cloning of xlProminin-1, 2 and 3 cDNAs.

**Targets**	**Sequences (5′-3′)**	**Purpose**
xlProminin-1	CGGGGTACCGTAGATCTCTTGGCATTCGCTAATG	RT–PCR
	CGCGGATCCTTATGATAACCATTATTACCATTTTCCA	RT–PCR
	ATTGACTGCTCCAGTGGCAC	5′RACE outer primer
	CATTAGCGAATGCCAAGAGATCTAC	5′RACE inner primer
	GTTTGGTTTGGGAGGAGCAACTGTG	3′RACE outer primer
	ATGGACACCGAAGATGTTTATGATG	3′RACE inner primer
xlProminin-2	CAGAAGGTCCAAGAGGAGTTTGC	RT–PCR
	CCAGGGATTAGTTATATTGTCACACA	RT–PCR
	ATTTGCATCGACTACAGCCTGC	5′RACE outer primer
	TTTTCAAGCACCAGGGGAAGGC	5′RACE inner primer
	TTGGAAAACCTGGCAACCGTGC	3′RACE outer primer
	AACTCCTTGGCTGAACTGGTCC	3′RACE inner primer
xlProminin-3	CATGTTGGCTGGAAATATCTGCATGTT	RT–PCR
	TTGCCGAGATGTTGTCATCTCCAT	RT–PCR
	TAGTAGCCACCAGGAGCTTAGC	5′RACE outer primer
	TCACGGCATTTACGGTGTTCAC	5′RACE inner primer
	AATTTTGCTCGCTGTGGACCAGTTGCCAGA	3′RACE outer primer
	TGCAAAGCACTACAGGAGGATGAAGACTTCT	3′RACE inner primer
Adaptor or anchor primers for RACE	GGCCGCACGCGTCGACTAGTACGGGIIGGGIIGGGIIG	5′RACE adaptor primer
	GGCCGCACGCGTCGACTAGTAC	5′RACE anchor primer
	CGACTCACTATAGGGCGATTTTTTTTTTTTTTTTT	3′RACE adaptor primer
	CGGGGTACCGTAATACGACTCACTATAGGGCGA	3′RACE anchor primer

### Cloning of *Xenopus laevis* prominin-2 and 3 cDNAs

We performed BLAST searches with sequences of known prominin homologs, including human prominin-1 (NP_006008) and 2 (NP_653308), and mouse prominin-1 (NP_032961) and 2 (NP_620089), against the Ensembl genomic database of *Xenopus tropicalis* [41]. Partial sequences resulting from this search were used to search the *X. laevis* EST databases at the Sanger Institute and to design primers to amplify prominin homologs from *X. laevis* by RT–PCR and RACE, using cDNA synthesized from *X. laevis* retina or testis total RNA as templates. Sequences of primers used in RT–PCR and RACE are provided in Table 1.

### Sequence analysis of prominin homologs

Maximum likelihood phylogeny of prominin homologs from different species was generated using PhyML 3.0 software [42]. cDNAs of prominin homologs of the human, chimpanzee, rhesus monkey, rat, mouse, horse, cattle, dog, zebrafish, nematode, and fly were retrieved from GenBank. Prominin homologs of *X. tropicalis* were cloned by methods similar to those used for *X. laevis*; cDNAs synthesized from *X. tropicalis* retina or testis total RNA were used as templates for PCR and RACE. Bovine and platypus prominin homologs were predicted from genomic sequences by BLAST search and by aligning them with cDNA sequences of known prominin homologs and searching for the consensus exon/intron boundaries [43,44]. Platypus genomic sequences were retrieved from Ensembl Ornithorhynchus anatinus version 60.1n (OANA5) Chromosome 4. Bovine genomic sequences were retrieved from Ensembl Bos taurus version 60.4i (Btau_4.0) Chromosome 6. Other software used in sequence analysis included ClustalW for sequence alignments [45], SignalIP 3.0 for signal peptide prediction [46], NetOGlyc 3.1 for glycosylation site prediction [47], TMHMM Server, version 2.0 for prediction of transmembrane domains [48,49], and ScanProsite software for identification of PDZ-binding motifs [50].

### RNA isolation and cDNA synthesis

RNA was extracted from the adult *X. laevis* retina, brain, testis, and kidney using TRIzol reagent (Invitrogen) and used to synthesize first-strand cDNAs with SuperScript II reverse transcriptase (Invitrogen) and oligo-dT primers (Amersham, Piscataway, NJ) according to protocols supplied by the manufacturers.

### Analysis of alternative splicing

Alternatively spliced exons of xlProminin-1, 2, and 3 were identified by sequencing and alignment of multiple cDNA clones. Exon/intron boundaries were determined by comparing cDNA sequences with *X. tropicalis* genomic DNA sequences. The reference genomic sequences used were: *X. tropicalis* unplaced genomic scaffold, v4.2 XENTRscaffold_393, whole genome shotgun sequence, containing the prominin-1 gene, GenBank accession NW_003163719; *X. tropicalis* unplaced genomic scaffold, v4.2 XENTRscaffold_30, whole genome shotgun sequence, containing the prominin-2 gene, GenBank accession NW_003163356; *X. tropicalis* unplaced genomic scaffold, v4.2 XENTRscaffold_640 and 3098, whole genome shotgun sequence, containing the prominin-3 gene, GenBank accession NW_003163966 and NW_003166412, respectively. Alternative splicing at the C-terminus of xlProminin-1 in the retina, brain, testis, and kidney was examined by amplifying a cDNA fragment containing this region by RT–PCR. Primers used in this analysis were: 5′-ATA AGA ATG CGG CCG CCT GCA GAG AAC ATC CTT TGA TAT CGA G-3′ and 5′-CGC GGA TCC CAG ATT ATT TTC TGA AAG TCA TTT CTC C-3′. (Note that the Not I and BamH I sites were incorporated into the primer sequences to facilitate cloning.) The profiles and relative abundances of differentially spliced transcripts were analyzed by comparing the sizes and intensities of RT–PCR products resolved by electrophoresis on a 1% agarose gel. Optical densities were measured with ImageJ software [51] for quantification of different splicing isoforms of xlProminin-1. Individual bands were extracted from the gel using the GeneClean® III Kit (Qbiogene, Irvine, CA), cloned into the pCR 2.1 TOPO cloning vector (Invitrogen) and sequenced.

### Semiquantitative reverse-transcription PCR

Two primer pairs were designed for specific amplification of each prominin homolog (Table 2 for sequences of primers). Reverse-transcriptase PCR amplification of a fragment of β-actin was used as a loading control to establish that an equal amount of starting materials was used (Table 2). PCR reactions were assembled using an Advantage® 2 PCR Kit (Clontech, Mountain View, CA) in a final volume of 100 μl containing 4 μl of cDNA. Aliquots (20 μl) taken at the end of the 27th, 31st, 35th, and 39th cycles were visualized on a 1% agarose gel by ethidium bromide staining, and digital images were analyzed using Image J software. The total amount of DNA in selected areas was represented with integrated optical densities. Results obtained with two different primer pairs were averaged and plotted.

**Table 2:** Primers used in semiquantitative reverse-transcription (RT)-PCR of xlProminin homologs.

**Targets**	**Primer pairs**	**Sequences (5′-3′)**	**Expected MWs**
xlProminin-1	1	GAAATTGGCTTTATCATTGCGGCAGTA	1939-1993^a^ bps
		AGTAGCCAATGACTTCATCCATTAACTT	
	2	AACCTTACCAACCAGATTAGAGGATCA	1601-1655^a^ bps
		ACCTTCCCCTCGATATCAAAGGATG	
xlProminin-2	1	CAGAAGGTCCAAGAGGAGTTTGC	1800 bps
		CCAGGGATTAGTTATATTGTCACACA	
	2	GTGCTTGAAAAGATTGTATCTTCTTTC	1370 bps
		AAGGCATTGGCTTCACTCTGTAGC	
xlProminin-3	1	GAAGTTGACTTAATAATTGGCCAAAGT	1881 or 1902^b^ bps
		GAGAGCAATGAGAGAGACATTTCAG	
	2	CATGTTGGCTGGAAATATCTGCATGTT	1436 bps
		AACTGTCTTTCGCCAGCGTAGC	
*X. laevis* β-actin (as control)		GGCTATGCTCTACCACATGCCA	532 bps
		TGGAGCCACCAATCCAGACA	

^a^The expected MWs of these fragments varies with the presence or absence of the alternatively spliced exons 8a (24 bps), 11a (12 bps) and 19 (18 bps). The differences in sizes of these PCR products are too small to be resolved on a 1% agarose gel.

^b^The expected MWs of these fragments varies with the presence or absence of the alternatively spliced exons 24a (21 bps). The differences in sizes of the two PCR products are too small to be resolved on a 1% agarose gel.

## Results

### Identification of three prominin homologs in *Xenopus laevis*

**Figure 1:**
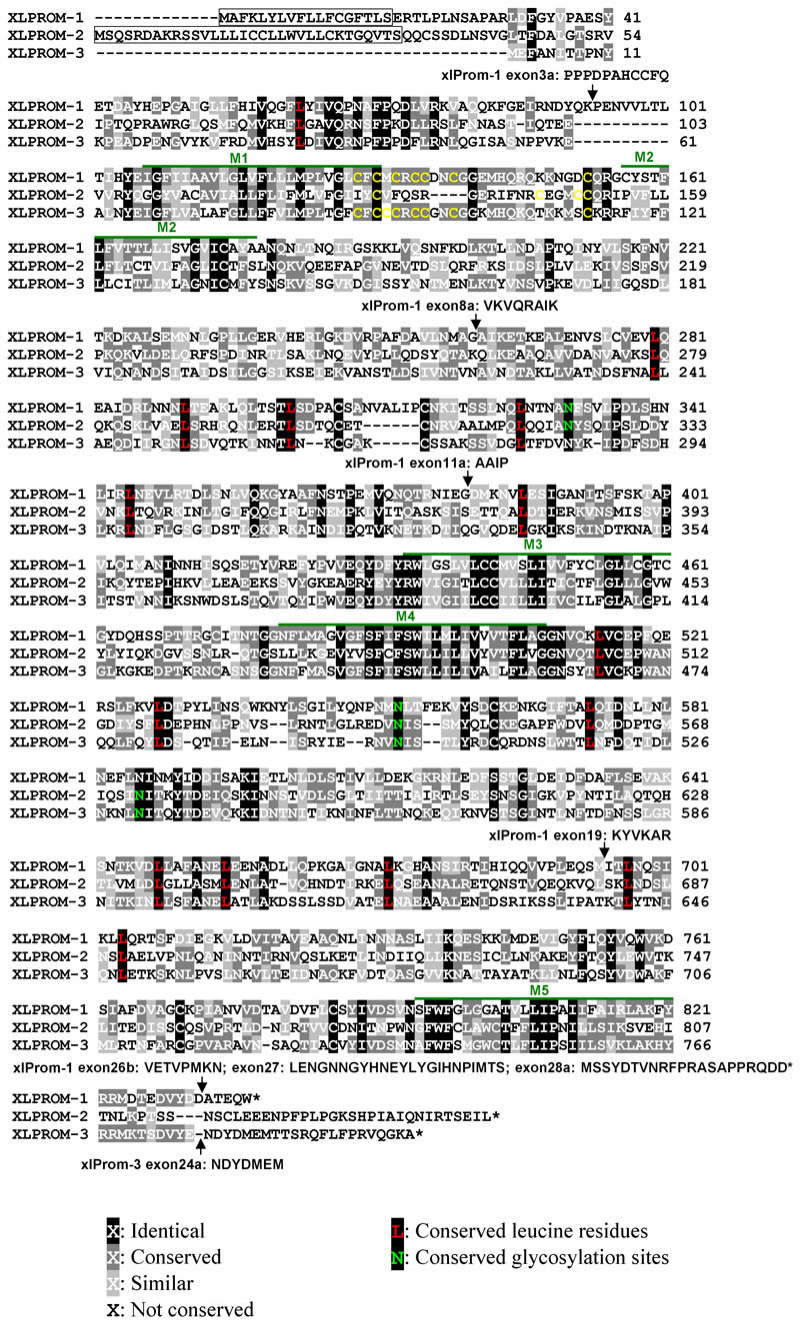
Alignment of the predicted protein sequences of three *X. laevis* prominin homologs, showing characteristic features of prominins, including the pentaspan transmembrane topology, a conserved cysteine rich domain, conserved leucine residues and N-glycosylation sites. We designated the three *X. laevis* prominin homologs as xlProminin-1, 2, and 3, respectively. Identical and conserved residues are indicated by differentially shaded boxes. Predicted transmembrane domains are marked with M#. The predicted signal peptides of xlProminin-1 and 2 are boxed. No signal peptide was predicted for xlProminin-3. Positions of alternatively spliced exons are indicated by arrows and the sequences of the alternatively spliced exons are given. Note that the N- and C-termini of the three *X. laevis* prominin homologs are less similar than other regions. Cysteine residues at the junction of M1 and the first intracellular domain (I1) are marked with yellow. Conserved leucine residues of the three xlProminin homologs are marked with red. Conserved glycosylation sites are marked with green.

By performing a BLAST search of the *X. laevis* EST database, we retrieved a sequence (GenBank accession: BJ061435) with 62% similarity to human prominin-1. BLAST search of the NCBI nucleotide database and the Ensembl *X. tropicalis* genomic database using the available sequences of prominin homologs from different species as query sequences revealed three different prominin homologs in *X. tropicalis*. These sequences have the assigned GenBank accession numbers of BC127277, XM_002932561, and XM_002940570. Sequence BC127277 showed a high degree of protein sequence identity (95%) to the above mentioned *X. laevis* sequence BJ061435. However, *X. laevis* homologs of sequences XM_002932561 and XM_002940570 could not be retrieved from any public cDNA or genomic database by BLAST search, perhaps due to incomplete coverage of the *X. laevis* sequences by those databases. They were instead cloned by RT–PCR using specific primer pairs based on *X. tropicalis* prominin homolog sequences, employing cDNA synthesized from total RNA isolated from *X. laevis* tissues as templates. Multiple clones were obtained and sequenced. Full-length cDNA sequences of the three *X. laevis* prominin homologs were obtained by performing 5′ and 3′ RACE. Predicted protein sequences are aligned in Figure 1.

### Phylogenetic analysis of prominin homologs

**Figure 2:**
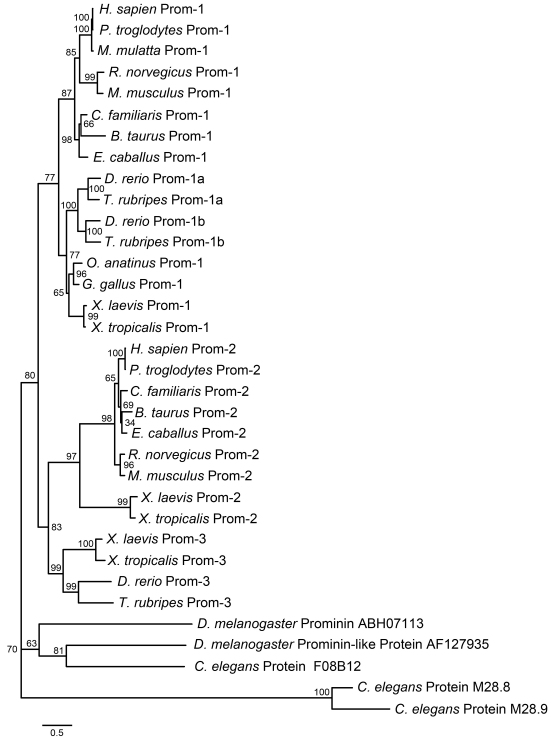
Phylogenic analysis of prominin homologs from several species. This phylogenetic tree was constructed to show the existence of multiple prominin homologs in metazoan animals from fly to human and their evolutionary relationship. Three *X. laevis* prominin homologs, designated as xlProminin-1, 2, and 3, are placed in branches of the consensus phylogenetic tree with prominin-1, 2, and 3 from other species. The numbers at each node are the percentage bootstrapping values of 100 replicates. The evolutionary distance of any two sequences is represented by the length of the branches. Sequences used in the phylogenetic analysis are excerpted from: *H. sapien* Prom-1 (NP_006008), *P. troglodytes* Prom-1 (XP_517115), *M. mulatta* Prom-1 (NP_001070888), *R. norvegicus* Prom-1 (NP_068519), *M. musculus* Prom-1 (NP_032961), *C. familiaris* Prom-1 (XP_850831), *B. taurus* Prom-1 (HQ159409), *E. caballus* Prom-1 (XM_001498729), *D. rerio* Prom-1a (NP_001108615), *T. rubripes* Prom-1a (HQ159405), *D. rerio* Prom-1b (NP_932337), *T. rubripes* Prom-1b (HQ159406), *O. anatinus* Prom-1 (HQ159408), *G. gallus* Prom-1 (XP_001232165), *X. laevis* Prom-1 (XM_001163920), *X. tropicalis* Prom-1 (HQ159400), *H. sapien* Prom-2 (NP_653308), *P. troglodytes* Prom-2 (XP_001143498), *C. familiaris* Prom-2 (XP_854455), *B. taurus* Prom-2 (XP_599188), *E. caballus* Prom-2 (XP_001494215), *R. norvegicus* Prom-2 (AAN63818), *M. musculus* Prom-2 (NP_620089), *X. laevis* Prom-2 (NP_001163922), *X. tropicalis* Prom-2 (HQ159402), *X. laevis* Prom-3 (NP_001163921), *X. tropicalis* Prom-3 (HQ159403), *D. rerio* Prom-3 (XP_684527), *T. rubripes* Prom-3 (HQ159407), *D. melanogaster* Prominin (ABH07113), *D. melanogaster* Prominin-like protein AF127935 (NP_647770), *C. elegans* protein F08B12 (NP_509907), *C. elegans* protein M28.8 (NP_496294), *C. elegans* protein M28.9 (NP_496296).

Previous taxonomic studies of prominins have divided prominin homologs into three groups. These are prominin-1 [2,31] and prominin-2 in mammals [3], and prominin-3 in zebrafish [52]. Two variants of prominin-1, namely prominin-1a and 1b, were identified in zebrafish [52]. We performed protein phylogenetic analysis using the PhyML 3.0 software to compare *X. laevis* prominin homologs with available prominin sequences; the results are presented in Figure 2. The sequences used to construct the phylogenetic tree were from online public databases, newly cloned cDNAs, or predicted mRNAs from genomic sequences (Methods). Since the N- and C-termini of prominin homologs were incomplete in many of the retrieved sequences, only the sequences from exon 4 to exon 24 of prominin-1 and homologous sequences of other prominins were used for the phylogenetic analysis. The analysis revealed that the first cloned prominin homolog is the *X. laevis* ortholog of prominin-1, and is therefore designated as xlProminin-1. The other two prominin-like cDNAs found in *X. laevis* were named xlProminin-2 and xlProminin-3 according to their positions on the phylogenetic tree.

All newly cloned and predicted prominin homologs were also named according to their positions on the phylogenetic tree, and full sequences (including certain splice variants) were deposited in GenBank with assigned accession numbers (see Table 3 and Table 4), including sequences from: *X. tropicalis*, fugu, platypus, and cattle. We made new predictions for bovine prominin-1 from genomic sequences, because previously predicted bovine prominin-1 sequences (XP_875477 and XP_002688513) contain obvious errors and cannot be used for an accurate phylogenetic analysis. Our newly predicted bovine prominin-1 was assigned with the GenBank accession HQ159409. Sequences used in the phylogenetic analysis and the alignment of these sequences are presented in Appendix 1.

**Table 3:** Evidence for existence of full-length or partial sequences of prominin homologs in non-mammalian vertebrates and platypus.

**Species**	**Prominin-1**		**Prominin-2**	**Prominin-3**
**Prominin-1a**	**Prominin-1b**
Zebrafish (*D.rerio*)	NP_001108615	NP_932337	Not found	XP_684527
Fugu (*T. rubripes*)	HQ159405	HQ159406	Not found	HQ159407
Frog (*X. laevis*)	NP_001163920		NP_001163922	NP_001163921
Frog (*X. tropicalis*)	HQ159400, HQ159401, BC127277		HQ159402	HQ159403
Chicken (*G. gallus*)	XP_001232165		XP_425759	AF406812
Platypus (*O. anatinus*)	HQ159408		XP_001509143	XP_001511144

**Table 4:** Presence or absence of alternatively spliced exons in full-length cDNAs of the xlProminin-1 and xtProminin-1 splice variants.

**Sources**	**Splice variants**	***3a***	***8a***	***11a***	***19***	***26b***	***27***	***28a***	***X. laevis* (xlProminin-1)**	***X. tropicalis* (xtProminin-1)**
**Retina**	**s1**	-	-	-	+	-	-	-	JF274976	HQ159400
	**s2**	-	-	+	+	-	-	-	JF274977	
	**s3**	+	-	+	+	-	-	-	JF274978	
	**s4**	-	-	+	+	+	-	-	JF274979	
	**s5**	-	-	-	-	+	+	+	JF274980	HQ159401
**Kidney**	**s6**	+	-	-	+	+	+	+	FJ358518	
	**s7**	-	+	+	+	-	-	-	JF274981	

We compared the results of our phylogenetic analysis of prominin homologs with previous findings and found that *X. laevis* and *X. tropicalis* are the only species verified so far to possess all three prominin orthologs. Only two prominin homologs, prominin-1 and 2, were found in human and mouse. We conducted BLAST searches of the NCBI nucleotide database and the Ensembl database for prominin homologs in more species of mammals, including the human, chimpanzee, rhesus monkey, cattle, horse, dog, and rat. All retrieved prominin homolog sequences from these mammals were better grouped with prominin-1 or 2 than with prominin-3. A total of three prominin homologs were found in zebrafish (*Danio rerio*) and fugu (*Takifugu rubripes*). However, these fish do not have a prominin-2 homolog; they instead have two prominin-1 homologs, prominin-1a and 1b, and a prominin-3 homolog [52]. Incomplete sequences of prominin homologs were retrieved from EST or genomic databases of the chicken (*Gallus gallus*) and platypus (*Ornithorhynchus anatinus*, a primitive egg-laying mammal). GenBank accession numbers of these sequences are given in Table 3. They seem to group with prominin-1, 2, or 3 by sequence alignment. However, further taxonomic studies are needed to precisely position them in the phylogeny of prominins. Three *C. elegans* prominin homologs and two *D. melanogaster* prominin homologs were used in constructing the prominin phylogeny. These sequences did not belong to any of the three groups of prominins in our analysis, suggesting the possibility of other prominin homologs.

### All three prominin homologs are predicted to be pentatransmembrane glycoproteins

Similar to mammalian prominins, hydropathy analysis using TMHMM software predicted that all *X. laevis* homologs have five hydrophobic domains, which likely constitute membrane-spanning domains that divide the sequence into two small intracellular loops and two large extracellular domains. However, the last hydrophobic sequence in xlProminin-2 did not reach the set threshold of TMHMM for prediction of a transmembrane domain (Appendix 2). Alignment of translated protein sequences from three cloned *X. laevis* prominin homolog cDNAs reveals that the most conserved regions are the transmembrane domains, as illustrated in Figure 1. A cysteine-rich region at the junction of the first transmembrane domain and intracellular loop (M1 and I1) is conserved in xlProminin-1 and 3, but is less conserved in xlProminin-2. Multiple leucine residues on the extracellular domains (E2 and E3) are conserved in all three xlProminin homologs. The conserved cysteine-rich region and leucine residues were all previously identified in mammalian prominin-1 and 2 [3,6,53,54]. The extracellular domains of all three xlProminins contain consensus sequences for N-linked glycosylation. In contrast to mouse and human prominin-1, no PDZ-binding motifs were identified in the C-terminal splice variants of xlProminin-1, 2, or 3 using ScanProsite software. PDZ-binding motifs were identified on the C-termini of certain splice variants of prominin-1 in the rodent (s1, s2, s7, s8, and s11) and human (s3, s4, s5, and s9) models, [31] and could potentially modulate interactions of prominin-1 with other proteins.

### Three prominin homologs are differentially expressed in *Xenopus laevis* tissues

Although prominin-1 is broadly expressed, the principle phenotype observed in patients is retinal degeneration [19,20,22-24]. It was proposed that there may be functional and spatial overlap of prominin homologs in most tissues, such that they compensate for one another if a loss of function mutation occurs [3]. We therefore analyzed expression of the three xlProminin homologs in different *X. laevis* tissues to assess their relative expression levels in those tissues. We were particularly interested in determining whether any prominin homolog is predominantly expressed or absent in particular tissues among the four we studied.

**Figure 3:**
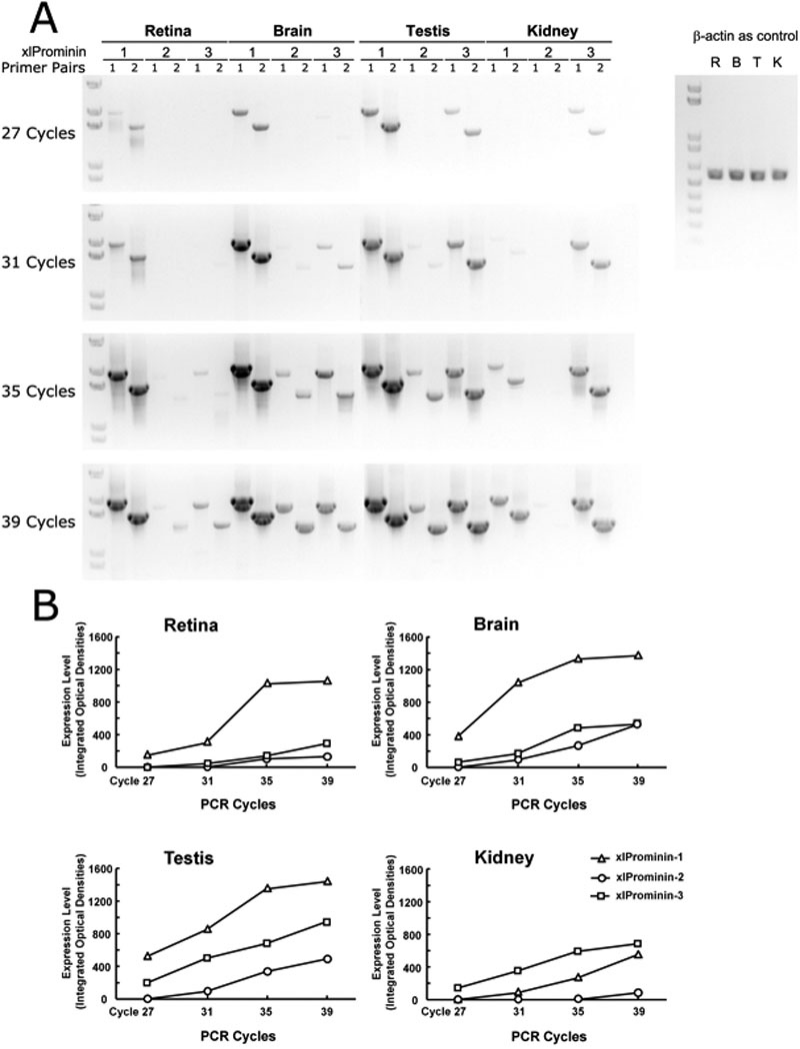
Semiquantitative reverse-transcription PCR analysis of xlProminin-1, 2, and 3 performed on the retina, brain, testis, and kidney. **A**: Aliquots (20 μl) of the PCR reaction were taken at the end of the 27th, 31st, 35th, and 39th cycles, separated and visualized on a 1% agarose gel. Two independent primer pairs (see Table 2) were used to amplify each xlProminin homolog in each tissue to ensure the specificity and efficiency of the PCR reaction. A fragment of *Xenopus laevis* β-actin cDNA was also amplified from equivalent starting material of total RNA to serve as a control. The Invitrogen 1KB PLUS DNA (Cat. No. 10787–018) ladder was used for size standards. **B**: Integrated signals in areas occupied by individual DNA bands were quantified and plotted to compare the relative expression level of xlProminin-1, 2, and 3 in four *X. laevis* tissues: the retina, brain, testis, and kidney. xlProminin-1, 2 and 3 are expressed in all four tissues. Among the three prominin homologs, prominin-1 is predominantly expressed in the retina, brain, and testis, while prominin-3 is predominantly expressed in the kidney. The expression level of prominin-2 is the lowest of the three prominin homologs in all examined tissues, and is barely detectable in the kidney.

We performed semiquantitative RT–PCR with mRNAs isolated from the *X. laevis* retina, brain, testis, and kidney to analyze the expression profiles of xlProminin-1, 2, and 3, because these four tissues are mRNA rich and differ significantly in their function and metabolism. The results are presented in Figure 3. Precautions were taken to ensure specificity of the PCR reaction and accuracy of the analysis, including ensuring that measurements were consistent when using two completely different primer pairs for each prominin homolog. We also used relatively large PCR products (>1.4 kb), such that small nonspecific products could easily be recognized and disregarded. For the primer design, we carefully chose primers that only complement the desired homolog. We found that mRNAs of three xlProminin homologs were differentially expressed in these tissues: xlProminin-1 was the predominant form found in the retina, brain, and testis, while x1Promnin-3 was the predominant form found in the kidney. The expression level of xlProminin-2 was the lowest of the three prominin homologs in all examined tissues in this frog.

### Splice variants of *Xenopus laevis* prominin-1

**Figure 4:**
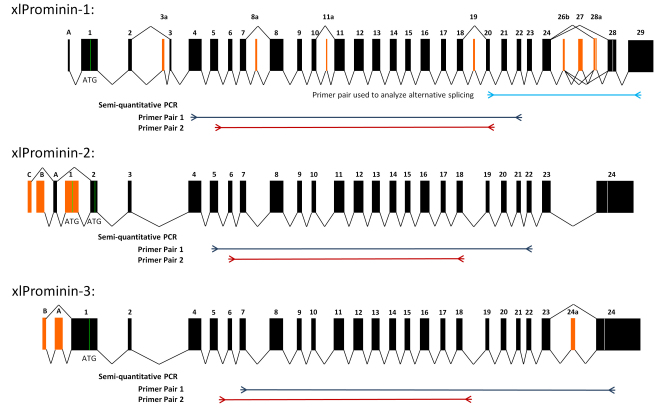
Exon organization of xlProminin-1, 2, and 3. Homologous exons of xlProminin-1, 2, and 3 are aligned. Genes of all three xlProminins are alternatively spliced and their exon organization is evolutionarily conserved. Constitutive exons are marked in black. Alternatively spliced exons are marked orange. Spliced forms identified in cDNA clones are indicated by joining lines. Translation start sites (ATG) are marked with green lines and the positions of translational stop codons are marked with white lines. Note the complex splicing of exons 26b, 27, and 28a, which generates several distinct isoforms of xlProminin-1. Positions of primers used in reverse-transcription (RT)-PCR for semiquantitative analysis of mRNA expression and for alternative splicing are indicated on the diagram.

A total of seven full-length splice variants (s1–s7) of xlProminin-1 were cloned and sequenced, and the presence or absence of certain alternatively spliced exons is summarized in Table 4. These splice variants were not named following the nomenclature for splice variants of human and mouse prominin-1 [31], because some alternative exons in xlProminin-1 do not have corresponding exons in human and mouse, and vice versa. Comparison of exon organization of *X. laevis*, mouse, and human prominin-1 is described in detail below. Alignment of these cloned cDNAs reveals that there are at least seven alternatively spliced exons in the xlProminin-1 gene (Figure 4). We numbered exons following the established nomenclature [31]. Exons 3a (“a” for “alternative”), 8a, 11a, and 28a are newly discovered alternative exons found in *X. laevis*. They were numbered based on their positions relative to the constitutive exons. Exons 19, 26b, and 27 are alternative exons in the human and mouse that were alternatively spliced in *X. laevis*. Alternatively spliced exons 26b to 28a of xlProminin-1 reside in a region encoding the C-terminus of the protein. Exons 3 and 28 of xlProminin-1 were always retained, despite the fact that they appear to be alternative in the mouse and human [2,31]. We noticed that the inclusion or deletion of these alternatively spliced exons does not change the downstream reading frame, except for exon 28a, within which an in-frame stop codon resides. Inclusion of exon 28a results in a longer but different protein sequence for the C-terminus of xlProminin-1 relative to other isoforms.

### *Xenopus laevis* prominin-2 and 3 are also alternatively spliced

Multiple variants arising from alternative splicing of all three prominin homologs were identified among mRNAs isolated from different tissues (Figure 4). A region near the 5′ end of xlProminin-2 transcript was alternatively spliced. Exon 1 of xlProminin-2 was predicted to contain the translation initiation site (ATG). Surprisingly, exon 1 was skipped in one of the sequenced clones, implying an alternative start codon in exon 2. This proposed alternative start codon was associated with a suboptimal Kozak consensus sequence (−3 CAAATGG +4). The sequence of one full-length xlProminin-2 was obtained; this was assigned GenBank accession FJ358519. The 5′ end of xlProminin-3 transcript and its exon 24a were found to be alternatively spliced. Two full-length splice variants (s1 and s2) of xlProminin-3 were obtained, and assigned GenBank accession numbers FJ358520 and JF274982, respectively. xlProminin-3.s1 contains an alternatively spliced exon 24a, while xlProminin-3.s2 does not. Details of exon organization, alternative 5′ untranslated region (UTR) or alternative polyA sites at 3′ UTR of xlProminin-1, 2, and 3 are provided in Appendix 3. The functional significance of the alternatively spliced variants is unknown.

### Characterization of alternative splicing of *Xenopus laevis* prominin-1 in exons 20 to 28

**Figure 5:**
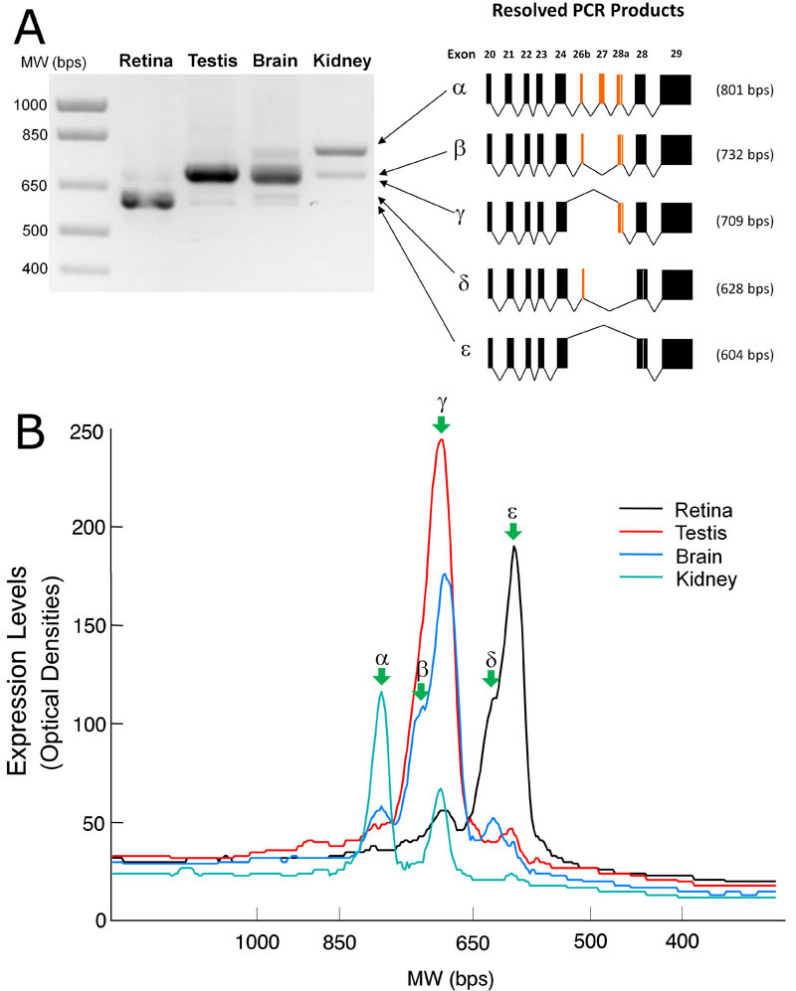
Analysis of the xlProminin-1 alternatively spliced isoforms in four tissues: the retina, brain, testis, and kidney. We found that profiles of the xlProminin-1 alternative splicing are different in these tissues. **A**: Products of reverse-transcription PCR (RT–PCR) from a region of extensive alternative splicing on the xlProminin-1 gene (exon 20 to 28) were separated on a 1% agarose gel and visualized with ethidium bromide staining. Five discrete PCR products were excised from the gel, cloned, and sequenced. Their exon compositions were determined and each product was designated with a Greek letter (α, β, γ, δ, and ε) for identification when used in the quantitative analysis shown in panel B. The predominant isoforms of xlProminin-1 expressed in the retina lack the alternatively spliced exons 26b, 27, and 28a, whereas the predominant isoform of xlProminin-1 expressed in the kidney retains these exons. The predominant isoforms of xlProminin-1 expressed in the brain and kidney retain exon 28a. Exon 27 was retained only when exon 26b was retained as well. **B**: Quantification of the xlProminin-1 alternatively spliced isoforms in four tissues: the retina, brain, testis, and kidney. The resolved RT–PCR products were linearly scanned and the optical densities were plotted. Green arrows indicate peaks that represent isoforms resulting from alternative splicing. The major isoform of xlProminin-1 expressed in the retina, ε, lacks the alternatively spliced exons 26b, 27, and 28a, whereas the major form of xlProminin-1 expressed in the kidney, α, retains all possible exons. The majority of xlProminin-1 expressed in the brain and kidney retains exon 28a.

Fargeas et al. previously reported that splicing of the prominin-1 gene in mice results in great variations in the sequences of alternative C-termini [32]. We therefore examined the splicing profile of xlProminin-1 within a region from exon 20 to 28 by RT–PCR (Figure 5). We found that the two most abundant isoforms of xlProminin-1 in the *X. laevis* retina did not contain exon 27; their exon compositions were: (20–24)+26b-27-28a-28+ (ε in Figure 5) or (20–24)+26b+27-28a-28+ (δ in Figure 5). Isoforms containing exon 28a were also detected in the retina; their exon compositions were: (20–24)+26b-27-28a+28+ (γ in Figure 5), (20–24)+26b+27-28a+28+ (β in Figure 5) and (20–24)+26b+27+28a+28+ (α in Figure 5). However, isoforms containing exon 28a only accounted for a very small proportion of all xlProminin-1 in the retina (Figure 5). This result was unlikely to be biased by preferential amplification of smaller PCR products, since profiling of xlProminin-1 alternative splicing in this region gave very different results in the kidney, testis, and brain: Those tissues preferentially express the isoforms with exon 28a (Figure 5).

### Comparison of the exon organization of *Xenopus laevis*, mouse, and human prominin-1

**Figure 6:**
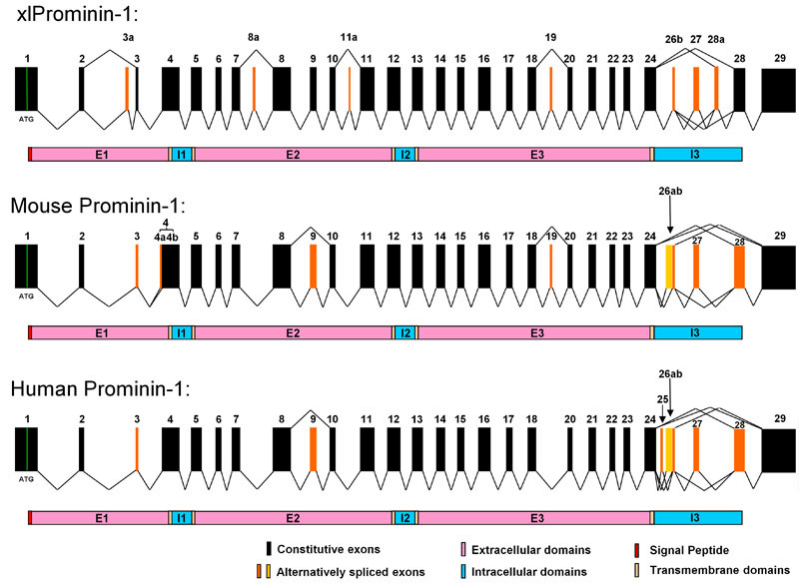
Comparison of exon organization of prominin-1 gene from *X. laevis*, mouse, and human. It appears that the gene structure of prominin-1 is evolutionarily conserved in these animals. Alternative splicing of the prominin-1 gene is seen in all three animals, however, with considerable differences in the choices of alternative exons and the splicing patterns. Homologous exons of xlProminin-1 and mouse and human prominin-1 are aligned for comparison of their exon organization. Constitutive exons are marked in black. Alternatively spliced exons are marked in orange. Spliced forms identified in cDNA clones are indicated by jointed lines. Note that homologous exons are assigned with the same number to maintain consistency with preexisting nomenclature [2,31]. Exons 26b and 27 are conserved and alternatively spliced in all three species. Alternative exons 3a, 8a, 11a, and 28a of xlProminin-1 are not found in mouse or human prominin-1. Splicing of alternative exons 4a results from using an alternative 3′ splice site in exon 4, and is only observed in mouse prominin-1 [31]. Alternative exon 19 of xlProminin-1 is alternatively spliced in mouse prominin-1, but no evidence has been found that this exon is alternatively spliced in human prominin-1. Alternative exon 25 of human prominin-1 is not found in prominin-1 from the mouse or *X. laevis*. The alternative exon 26a of mouse prominin-1 and the alternative exons 25 and 26a of human prominin-1 are not found in xlProminin-1. Exons 3, 9, and 28 of mouse prominin-1 are alternative, but appear to be constitutive in *X. laevis*. The region encompassing exons 24 to 28 of xlProminin-1 and homologous sequences of the prominin-1 gene from the mouse and human are regions of extensive alternative splicing. Diagrams of translated proteins are aligned with their coding sequences. Divisions of proteins by predicted transmembrane domains are marked.

The organization and splicing of prominin-1 exons in the mouse and human have been well characterized [30-32]. To determine whether the gene structure is conserved in xlProminin-1, we aligned all identified exons of prominin-1 from *X. laevis*, mice, and humans (Figure 6). Some exons found in one species were absent in another, but these were always alternatively spliced exons (Figure 6). We also correlated the positions of all alternatively spliced exons of xlProminin-1 to its membrane topology and found that all alternatively spliced exons represent sequences coding for the extracellular domains (E1, E2, and E3) or the C-terminus of the protein, as was previously observed in prominin-1 from mouse and human [3,30-32].

## Discussion

All members of the prominin family are predicted to be pentaspan transmembrane proteins with extracellular N-termini and intracellular C-termini [1,3,7,55]. They have two large extracellular domains (around 250 amino acids each) and two small intracellular domains (around 30 amino acids each). The two extracellular domains are predicted to be N-glycosylated and have conserved leucine residues. The first intracellular domain is unusually rich in cysteine (there are 7, 4, and 8 cysteine residues in the I1 domain of xlProminin-1, 2, and 3, respectively). These features are highly conserved in all identified prominin homologs, including xlProminin-1, 2, and 3 (Figure 1), suggesting that they are functionally important. However, the cysteine-rich domain of xlProminin-2 is not as well conserved as that of xlProminin-1 and 3.

Only two prominin homologs, namely prominin-1 and 2, were found in the human and mouse in previous studies [3,6,8]. Prominins in other mammals are less studied, but consistent with the findings in the human and mouse, we did not find prominin-3 homologs in the chimpanzee, rhesus monkey, horse, cattle, dog, or rat. By contrast, three prominin homologs were identified or their existence was inferred in some nonmammalian vertebrates (Table 3 and Table 4), including fish (*D. rerio* [52] and *T. rubripes*), frogs (*X. laevis* and *X. tropicalis*), and birds (*G. gallus*). However, phylogenetic analysis revealed that zebrafish and fugu lack an ortholog of prominin-2. These fish instead have two prominin-1 genes, namely prominin-1a and 1b [52]. In contrast, we determined that both *X. laevis* and *X. tropicalis* have all three prominin homologs that are orthologs of prominin-1, 2, and 3 (Figure 2). It appears that the third homolog was lost during mammalian evolution. Interestingly, genomic sequences encoding three prominin homologs are retrievable from the platypus (*O. anatinus*; Table 3). Studies of platypus prominin may provide clues for how prominin homologs have evolved. It may be that prominin-1 and 2 have extensive functional overlap, since there is shared expression of prominin-1 and 2 in most mouse tissues except the eye [3]. Our results showed that mRNAs of all three xlProminin homologs are expressed in the four examined tissues of *X. laevis* at various levels (Figure 3). The localization and functions of prominin homologs may overlap, and thus the loss of one homolog through mutation may be compensated for in most tissues by other prominins.

Several prominin homologs have been identified or predicted in *C. elegans* (e.g., NP_509907, NP_496294, and NP_496296) and *D. melanogaster* (e.g., NP_647770 and ABH07113) [1,6,56,57], while no prominin homologs have been found in bacteria, fungi, or plants, suggesting that the prominins appeared during metazoan evolution, increased to three genes in nonmammalian vertebrates, and subsequently reduced to two in mammals. Since prominins are not present in any unicellular species whose genome has been sequenced, we postulate that prominin-like proteins are important for intercellular interactions, such as cell-cell adhesion, migration, and communication, or in developmental processes. For example, Zelhof et al. demonstrated that in the *Drosophila* retina, prominin interacts with an extracellular matrix molecule, spacemaker, to control the apposition of photoreceptor rhabdomeres [57]. Similar to those described in Bardet-Biedl syndrome [58], polydactyly phenotypes observed in PROML1_1878_ (now designated as c.1841delG) patients [19] suggest a role in development: Mutations may directly or indirectly interfere with ciliary signaling pathways such as those mediated by smoothened [59].

Our study specifically addresses the mRNA expression of prominin homologs in *X. laevis*. The processes of mRNA and protein expression are often not well correlated. mRNAs may be regulated independently of the encoded proteins, and some mRNAs are not translated at all. Such genes are categorized as “pseudogenes” [60]. In fact, nontranslated mRNAs can serve important roles in regulating the expression and degradation of other homologous mRNAs [61-64]. We have verified that the protein encoded by the xlProminin-1 gene is detectable in *X. laevis* tissues by both immunoblotting and immunohistochemistry (data not shown). To date, we have been unable to determine whether proteins of xlProminin-2 and 3 are synthesized in frog tissues. Nevertheless, the presence of long, uninterrupted, evolutionarily conserved reading frames (Figure 1) suggests that these do not represent pseudogenes.

We observed alternative splicing of all prominin homologs in *X. laevis* (Figure 4). Alternative splicing sites were not found in exons coding for the two small intracellular loops, but only in exons coding for the three extracellular domains and the C-terminus. A region encompassing exons 26b to 28a of the *X. laevis* prominin-1 gene is a region of extensive alternative splicing (Figure 5). In extreme cases, alternative splicing of prominin-1 genes can yield proteins with completely different C-termini, which can also vary greatly in length. The significance of these isoforms is unknown. Alternative splicing is regarded as a mechanism to diversify the mRNA products from a single gene, and can confer multifunctionality to gene products by generating isoforms that have novel protein interaction domains or localization signals. We found that certain xlProminin-1 splice variants are preferred in certain tissues; splicing variants with exon composition of 24+26b-27-28a-28+ and 24+26b+27-28a-28+ that encode proteins of shorter C-terminus length are the preferred isoforms in the *X. laevis* retina (Figure 5). It may therefore prove informative to examine the localization of alternatively spliced forms, or to search for novel interaction partners by techniques such as crosslinking, coimmunoprecipitation, or two-hybrid analysis. Tissue-specific distributions of certain isoforms could also explain the tissue-specific phenotypes (retinal degeneration) observed with mutations in this otherwise broadly expressed gene [19,20,22-24]. Comparison of exon organization of prominin-1 in the human, mouse, and frog reveals that although there are considerable differences in both the existence and composition of certain alternatively spliced exons, all three prominin-1 homologs share similar forms of alternative splicing, especially at the region encoding for the C-termini of the proteins (Figure 6). A recent study found that isoforms s11 and s12 that lack exon 27 are the predominant alternative splicing form in the human retina [24]. These findings support the use of *X. laevis* as a suitable model system for investigating prominin-1 function in the retina.

Our findings of three prominin homologs in *X. laevis* and characterization of their mRNA expression and alternative splicing paves the way for future studies of the function of these prominins in photoreceptors in this frog. There are many advantages of studying *X. laevis* photoreceptors in comparison to mammalian photoreceptors: The photoreceptors of *X. laevis* are much larger than mammalian photoreceptors, and are easily imaged in detail by confocal microscopy. This frog’s retina contains nearly equal numbers of rods and cones, allowing for comparative investigation of both cell types. Furthermore, studies of photoreceptor outer segment biosynthesis have been conducted in frogs because of their large size and high rates of membrane turnover [34,35]. Finally, transgenic manipulation of *X. laevis* is comparatively easy and allows for the study of the transgene effects in relatively large numbers of animals in a timely manner [36,37].

Future directions for studying the function of prominins in photoreceptors may be focused on the turnover, transport, subcellular localization, and interacting partners of these molecules in *X. laevis*. Disease-causing mutations of human prominin-1 could be introduced to xlProminin-1, if they reside in conserved domains, and used to develop useful transgenic *X. laevis* disease models. This approach has proven successful in many studies, including investigations of the pathogenesis of the rhodopsin P23H mutation [65-67] and T22N mutation of rab8 [68]. None of the currently identified mutations of prominin-1 that cause human disease are located in alternative exons. It is unclear if any of the alternatively spliced isoforms is associated with disease pathogenesis. It would be informative to elucidate the function of each specific splice isoform by the specific knockdown of its mRNA with the RNA interference technique [69,70], which has been successfully applied in *X. laevis* [71-73].

## Appendix 1. Sequences used in phylogenetic analysis.

To access the file, click or select the words “Appendix 1.” This will initiate the download of a pdf archive that contains the file.

## Appendix 2. Prediction of membrane topology of three xlProminin homologs.

To access the file, click or select the words “Appendix 2.” This will initiate the download of a pdf archive that contains the file.

## Appendix 3. Exon organization of the xlProminin genes.

To access the file, click or select the words “Appendix 3.” This will initiate the download of a pdf archive that contains the file.
